# The oral microbiome of patients with ischemic stroke predicts their severity and prognosis

**DOI:** 10.3389/fimmu.2023.1171898

**Published:** 2023-04-17

**Authors:** Wenbo Sun, Shengwen Huang, Xiaoli Yang, Yufan Luo, Luqiong Liu, Danhong Wu

**Affiliations:** ^1^ Department of Neurology, Shanghai Fifth People’s Hospital, Fudan University, Shanghai, China; ^2^ Department of General Medicine, Shaoxing People’s Hospital, Zhejiang University, Shaoxing, China

**Keywords:** RNA, ribosomal, 16S, *Veillonella*, saliva, ischemic stroke, oral health, microbiota

## Abstract

**Background and objectives:**

Stroke is a common group of cerebrovascular diseases that can lead to brain damage or death. Several studies have shown a close link between oral health and stroke. However, the oral microbiome profiling of ischemic stroke (IS) and its potential clinical implication are unclear. This study aimed to describe the oral microbiota composition of IS, the high risk of IS, and healthy individuals and to profile the relationship between microbiota and IS prognosis.

**Methods:**

This observational study recruited three groups: IS, high-risk IS (HRIS), and healthy control (HC) individuals. Clinical data and saliva were collected from participants. The modified Rankin scale score after 90 days was used to assess the prognosis of stroke. Extracted DNA from saliva and performed 16S ribosomal ribonucleic acid (rRNA) gene amplicon sequencing. Sequence data were analyzed using QIIME2 and R packages to evaluate the association between the oral microbiome and stroke.

**Results:**

A total of 146 subjects were enrolled in this study according to the inclusion criteria. Compared with HC, HRIS and IS demonstrated a progressive increase trend in Chao1, observed species richness, and Shannon and Simpson diversity index. On the basis of permutational multivariate analysis of variance, the data indicate a great variation in the saliva microbiota composition between HC and HRIS (F = 2.40, P < 0.001), HC and IS (F = 5.07, P < 0.001), and HRIS and IS (F = 2.79, P < 0.001). The relative abundance of *g_Streptococcus*, *g_Prevotella*, *g_Veillonella*, *g_Fusobacterium*, and *g_Treponema* was higher in HRIS and IS compared with that in HC. Furthermore, we constructed the predictive model by differential genera to effectively distinguish patients with IS with poor 90-day prognoses from those with good (area under the curve = 79.7%; 95% CI, 64.41%–94.97%; p < 0.01).

**Discussion:**

In summary, the oral salivary microbiome of HRIS and IS subjects have a higher diversity, and the differential bacteria have some predictive value for the severity and prognosis of IS. Oral microbiota may be used as potential biomarkers in patients with IS.

## Introduction

Stroke is a prevalent group of cerebrovascular diseases characterized by sudden onset of localized or diffuse brain dysfunction lasting longer than 24 h, which can lead to brain damage or death (1). Ischemic stroke (IS) constitutes approximately 87% of all stroke types and causes a significant disease burden globally due to the high prevalence and disability rate evaluated by the disability-adjusted life years (2–4). Despite public health education and standard management of stroke prevention, most patients with IS receive delayed diagnoses and have poor prognoses. Therefore, controlling the risk factors is essential in reducing the onset and severity of the stroke and improving patients’ prognosis (5). However, apart from recognized risk factors, some potential risks of stroke, such as periodontal infections, are gradually being discovered (6).

Syrjänen et al. first explored the relationship between oral health and cerebral infarction in 1989. The results show that severe dental infections were associated with male patients with IS (7). This report opens the prelude to studying the relationship between periodontitis and ischemic stroke. Over the past 30 years, several case-control studies, cross-sectional studies, and cohort studies have confirmed this association (8–12). A 15-year follow-up community cohort study of 10,362 stroke-free participants found an independent association between periodontal disease and stroke risk, and that regular oral periodontal care reduced the risk of stroke (13). Moreover, patients with IS with severe periodontitis have greater neurological deficits than periodontally healthy patients (14). Currently, accumulating research indicates that dysbiosis of oral bacteria is a primary focus in attempts to link stroke to periodontitis (15). Oral flora is the second most complex microbiota in the human body, only behind the gut (16). The Human Oral Microbiome Database measures approximately 150 genera and 700 species of bacteria (17). On the basis of 16S ribosomal RNA sequence technology, studies have shown that patients with periodontitis with a higher abundance of *Porphyromonas*, *Prevotella*, *Streptococcus*, *Spirochaetes*, *Synergistetes*, *Bacteroidetes*, *Aggregatibacter*, *Rothia*, and *Bacteroidaceae* in subgingival plaque and saliva compare with healthy individuals (18–20). Oral bacteria can release arginine-specific gingival proteases to promote the activation of platelets and lipid deposition, ultimately accelerating atherosclerosis (21, 22). A prospective observational study shows that oral bacteria of patients with stroke are diverse. However, no control group was established, so it cannot be speculated whether the change in microbiota occurred before or after stroke onset (23). However, no direct evidence reveals the correlation between the oral microbiome and stroke.

This study aims to describe the oral microbiota composition of IS, the high risk of IS, and healthy individuals and to profile the relationship between microbiota and IS prognosis. Our findings help predict the risk and prognosis of stroke and may reduce the harm of ischemic stroke to patients through specific interventions.

## Materials and methods

### Standard protocol approvals, registrations, and patient consents

This study was approved by the Ethics Committee for Human Experimentation of the Fifth People’s Hospital of Shanghai, China (approval number: KY2021-109 for Shanghai Fifth People’s Hospital). Before collecting data, patients or their guardians have written informed permission. All research procedures adhered to the tenets of the Declaration of Helsinki.

### Study population

**Figure 1:**
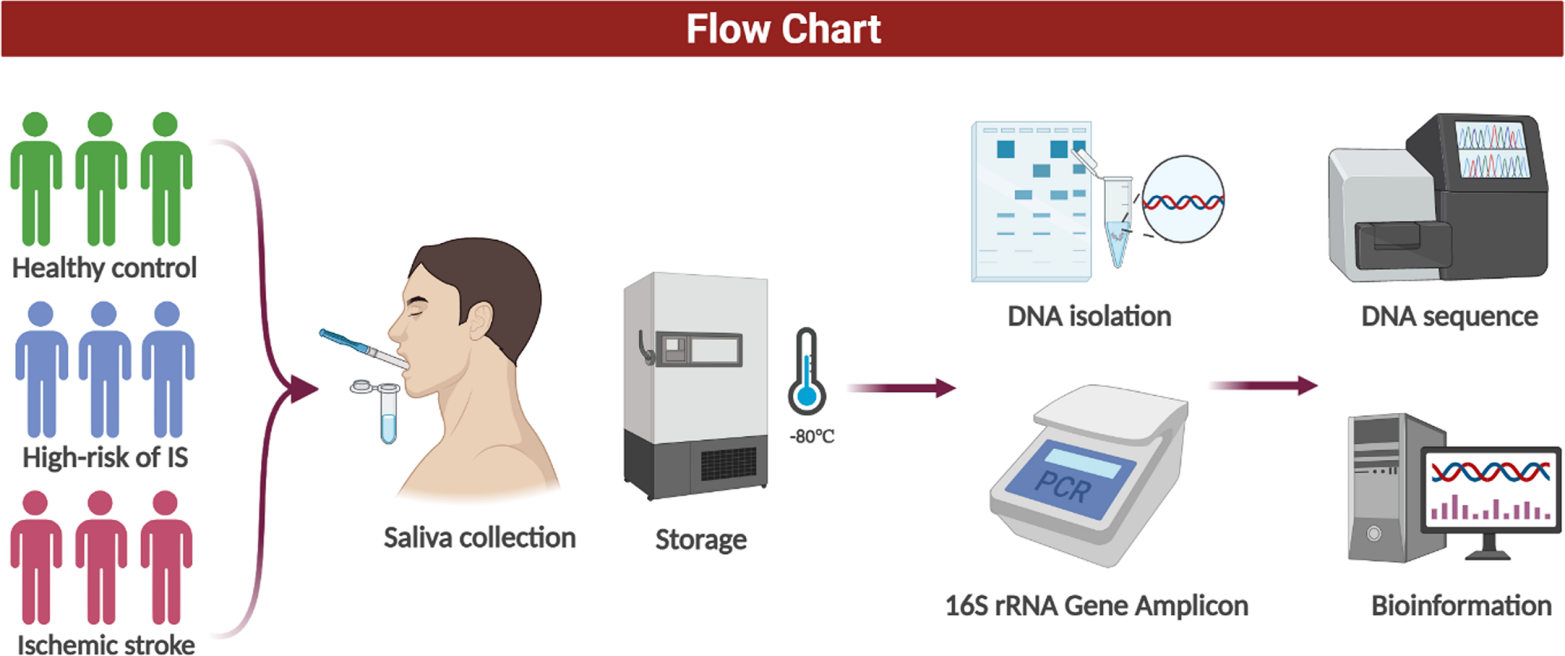
Flow chart.

This study was undertaken by the Neurology Department of the Shanghai Fifth People’s Hospital of Fudan University. We recruited three groups from January 2021 to November 2021: 1) patients with IS, 2) patients with high-risk IS (HRIS), and 3) healthy control (HC) individuals without cerebrovascular risk factors. Group 1 strictly adheres to the diagnosis criteria of the Trial of Org 10172 in Acute Stroke Treatment (TOAST) and included only large artery atherosclerosis (LAA) and small artery occlusion lacunar (SAA) of IS within 24 h of onset (24). For group 2, participants with at least three of the following risk factors for stroke were recruited: 1) history of hypertension; 2) history of diabetes; 3) history of valvular heart disease; 4) family history of stroke; 5) current smoking; 6) dyslipidemia: triglycerides (TGs) ≥ 2.26 mmol/L, total cholesterol (TC) ≥6.22 mmol/L, high-density lipoprotein (HDL) < 1.04 mmol/L, or low-density lipoprotein (LDL) ≥ 4.14 mmol/L; 7) exercise less than three times a week; and 8) body mass index (BMI) ≥ 26 kg/m^2^ (6). Subjects in the third group were recruited from the medical examination center of Shanghai Fifth People’s Hospital without cerebrovascular risk factors and were matched for gender and age to the first and second groups. Standardized questionnaires were used to obtain demographic and clinical data from all subjects, including gender, age, height, weight, history of past illness, family history, exercise, hematologic examination, smoking, and drinking history. In addition, patients who met the following criteria were excluded: history of stroke, myocardial infarction, heart failure, immune system disease, intestinal disease, cognitive impairment or severe liver dysfunction, and use of antibiotics and probiotics within 3 months. The content is shown in **Figure 1**.

### Severity and prognosis of stroke

The National Institutes of Health Stroke Scale (NIHSS) scores assessed stroke severity (25). According to neurologists’ consensus, the degree of neurological dysfunction in admission was separated into two grades: NIHSS score ≤ 4 and NIHSS score > 4. The modified Rankin scale (mRs) score was used to assess functional outcome 90 days following stroke onset, with 0–2 indicating favorable prognosis and 3–6 indicating bad outcomes. To measure the functional outcomes of patients with IS, we conducted telephone interviews with all patients 90 days following the original stroke (26).

### DNA extraction

Each participant used the spit method to collect 2 ml of fasting saliva in the morning and ensured they had not smoked gum or mouthwash within 3 h before sampling. Saliva samples were kept at −80°C until DNA extraction. We extracted DNA samples using the OMEGA Soil DNA Kit (M5635-02) (OMEGA BioTek, Norcross, GA, USA). NanoDrop NC2000 spectrophotometer (Thermo Fisher Scientific, Waltham, MA, USA) and agarose gel electrophoresis were used to evaluate the amount and quality of extracted DNA, respectively.

### 16S rRNA gene amplicon sequencing

Polymerase chain reaction amplification of the V3-V4 region of the bacterial 16S rRNA gene was performed by forwarding primer 338F (5′-ACTCCTACGGGAGGCAGCA-3′) and reverse primer 806R (5′-GGACTACHVGGGTWTCTAAT-3′). For multiplex sequencing, a sample-specific 7-bp barcode was inserted into the primers. Per-terminal 2 × 250-bp sequencing was performed using MiSeq Reagent Kitv3 on the Illumina MiSeq platform (Shanghai, China). According to the official tutorials, microbiome bioinformatics was performed with QIIME2 2019.4 (https://docs.qiime2.org/2019.4/tutorials/) (27). Primers were cut with the Cutadapt plugin after raw sequence data were demultiplexed using the Demux plugin. The DADA2 plugin was then used to quality filter, denoize, combine, and remove chimaera from the sequences. Non-singleton amplicon sequence variants (ASVs) were aligned with mafft and used to construct a phylogeny with fasttree2. Taxonomy was assigned to ASVs using the classify-sklearn naïve Bayes taxonomy classifier in the feature-classifier plugin against the Greengenes database (28).

### Bioinformatics and statistical analysis

Sequence data analyses were performed using QIIME2 and R packages (v3.2.0). ASV-level alpha diversity indices—such as Chao1 richness estimator, observed species, Shannon diversity index, Simpson index, and Faith’s phylogenetic diversity (PD)—were calculated using the ASV table in QIIME2 and visualized as box plots. ASV-level ranked abundance curves were generated to compare the richness and evenness of ASVs among samples. Beta diversity analysis was performed to investigate the structural variation of microbial communities across samples using UniFrac distance and visualized *via* principal coordinate analysis. Nested cross-validation (NCV) was used to provide predictions for all samples, with 10-fold cross-validation selected on the basis of the number of samples. A confusion matrix was used to visualize the predictions with heat maps and receiver operating characteristics (ROCs). The significance of the differentiation of microbiota structure among groups was assessed by permutational multivariate analysis of variance (PERMANOVA). Linear discriminant analysis (LDA) effect size (LEfSe) was performed to detect differentially abundant taxa across groups using the default parameter. SPSS 21.0 statistical software was applied for data analysis. For SSA and LAA groups, each demographic and clinical feature and normal distribution continuous variables were presented as mean ± standard deviation and compared using an independent sample t-test. The categorical variables are expressed in frequency (percentage) and are expressed in χ2 test or Fisher exact test, such as history of hypertension, diabetes, smoking, and family history of stroke. The Kruskal–Wallis H test was used for compare the means between the three groups, such as age, sex, BMI, and lipid level. Spearman’s rank correlation coefficient is used to analyze the relationship between oral microbiota and lipid metabolism.

## Results

One hundred forty-six subjects were enrolled in this study according to the inclusion criteria. The baseline data of subjects in each group are shown in **Table 1**. Saliva samples were obtained, and 16S rRNA sequencing was performed on 52 patients with IS [mean age, 57 ± 7.9 years; male patients, 30 (57.7)] and 48 HRIS [mean age, 58 ± 7.8 years; male patients, 25 (52.1)]. The severity and prognosis of IS were assessed using the NIHSS score on admission and the mRS score 90 days later. Forty-six gender- and age-matched HCs [mean age, 57 ± 8.6 years; male patients, 24 (52.2)] volunteered to donate saliva samples to the study. IS and HRIS have a higher level of TGs (p < 0.01), HDL cholesterol (p < 0.01), and LDL cholesterol (p < 0.05) than HC, but there was no significant difference in the level of TC.

**Table 1:** Characteristics of the study participants.

	Healthy controls	HRIS	IS	P-value
	n = 46	n = 48	n = 52	
Age, years	57 ± 8.6	58 ± 7.8	57 ± 7.9	0.657
Male, n (%)	24 (52.2)	25 (52.1)	30 (57.7)	0.812
BMI, kg/m^2^	24.7 ± 2.8	25.8 ± 4.3	25.7 ± 3.1	0.339
Current smoking, n (%)	**/**	16 (33.3)	22 (42.3)	0.412
Hypertension, n (%)	**/**	23 (47.9)	41 (78.8)	<0.01
Diabetes Mellitus, n (%)	**/**	13 (27.1)	24 (46.2)	0.063
Family history of stroke, n (%)	**/**	7 (14.5)	9 (17.3)	0.789
Triglycerides, mmol/L	1.30 ± 0.54	1.85 ± 0.63	1.79 ± 0.85	<0.01
Total cholesterol, mmol/L	4.45 ± 1.19	4.74 ± 1.08	4.83 ± 1.22	0.251
HDL cholesterol, mmol/L	1.43 ± 0.49	1.12 ± 0.45	1.06 ± 0.25	<0.01
LDL cholesterol, mmol/L	2.45 ± 0.85	2.98 ± 1.15	2.75 ± 1.05	0.042
NIHSS score > 4, n (%)	**/**	**/**	21 (40.4)	**/**
mRS score > 2, n (%)	**/**	**/**	16 (30.8)	**/**

BMI, body mass index; LDL cholesterol, low-density lipoprotein cholesterol; HDL cholesterol, high-density lipoprotein cholesterol.

### The composition of oral microbiota differs in three groups

**Figure 2:**
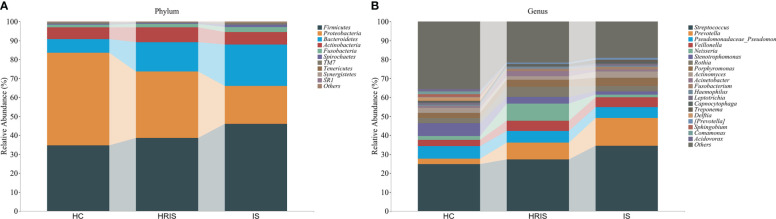
Relative abundance of oral microbiota at the phylum and genus level. **(A)** Top 10 relative abundance of oral microbiota at the phylum level. **(B)** Top 20 relative abundance of oral microbiota at the genus level.

**Figure 3:**
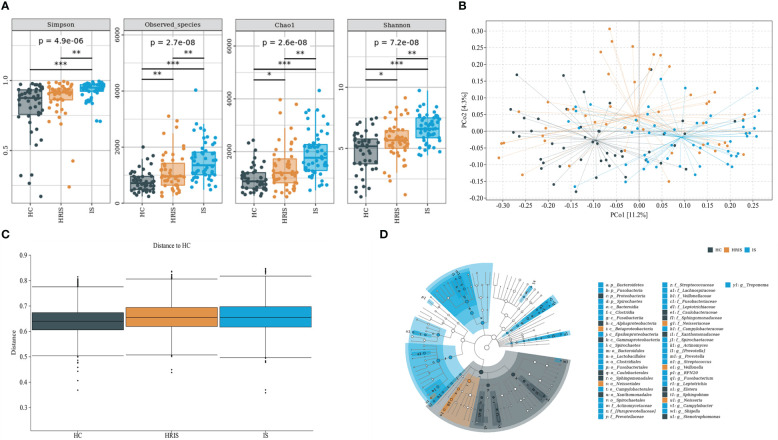
Oral microbiota composition of subjects with IS, HRIS and HC. **(A)** Comparison of α-diversity between the oral microbiota in three groups. **(B)** Principal coordinate analysis based on the unweighted UniFrac distances. **(C)** Unweighted UniFrac distances of each subgroup of control compared with HC. **(D)** linear discriminant analysis effect size-determined differential features.

This program identified 12 phylum and 112 genera of oral microbial by counting the number of taxonomic units in the ASV/Operational Taxonomic Units (OTU) tables. Oral microbiota with high relative abundance at phylum and genus levels in the cohort were displayed (**Figure 2**). In the HRIS group, the top nine with the highest relative abundance in the oral microbiota were *g_Streptococcus*, *g_Neisseria*, *g_Prevotella*, *g_Pseudomonadaceae*, *g_Rothia*, *g_Veillonella*, *g_Porphyromonas*, *g_Acinetobacter*, and *g_Actinomyces*. The 10 dominant genera in the oral flora of patients with IS were *g_Streptococcus*, *g_Prevotella*, *g_Pseudomonadaceae*, *g_Veillonella*, *g_Porphyromonas*, *g_Actinomyces*, *g_Rothia*, *g_Stenotrophomonas*, *g_Neisseria*, and *g_Fusobacterium* (**Figure 2B**). We adopt the Chao1 and observed species index to characterize richness and the Shannon and Simpson indices to characterize diversity to provide a more comprehensive assessment of alpha- diversity in oral microbiota. Compared with HC, HRIS and IS demonstrated a progressive increase trend in Chao1 and observed species richness, as well as Shannon and Simpson diversity indices (**Figure 3A**). We used the unweighted UniFrac distance to assess the beta diversity of the cohort. On the basis of PERMANOVA, the data indicate a great variation in the saliva microbiota composition between HC and HRIS (F = 2.40, P < 0.001), HC and IS (F = 5.07, P < 0.001), and HRIS and IS (F = 2.79, P < 0.001) (**Figures 3B, C**). Overall, our findings suggest that the oral microbiota of HRIS and IS are more diverse than that of HC and show an increasing trend.

**Figure 4:**
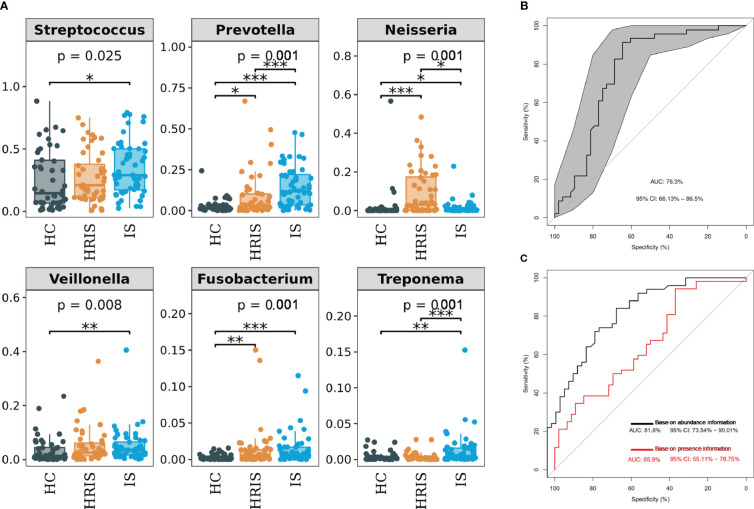
Differentiated bacteria of subjects with IS, HRIS and HC in genus level. **(A)** Comparison of α-diversity between the oral microbiota in three groups. **(B)** Machine learning for classification of HRIS and HC based on oral microbiota using the random forest algorithm. **(C)** Machine learning for classification of IS and HC based on oral microbiota using the random forest algorithm. *: P value ≤ 0.05; **: P value ≤ 0.01; ***: P value ≤ 0.001.

The oral microbiota composition of participants was characterized by LEfSe. Results show that the marker species and taxonomic hierarchy distribution of species from phylum to genus significantly enriched oral microbiota in LDA scores > 2 in the LEfSe analysis within each group. At the phylum level, the IS group had a significantly higher abundance of p_Bacteroidetes, p_Fusobacteria, and p_Spirochaetes than the other two groups (**Figure 3D**). We depict the differences in oral bacteria between the three groups at the genus level. The relative abundance of *g_Streptococcus*, *g_Prevotella*, *g_Veillonella*, *g_Fusobacterium*, and *g_Treponema* was higher in HRIS and IS compared with that in HC, and *g_Neisseria* of HRIS was the highest in the three groups (**Figure 4A**). To assess the value of microbiota alteration for screening people at high risk of IS, we compared the differences in bacteria between HC and HRIS and performed ROC analysis (the relative abundance of bacterial genus levels with LDA scores >2 was included in the model) (**Supplementary Figure 1**). The model’s area under the curve (AUC) was 76.3% (95% CI, 66.13%–86.5%; p < 0.01), which contributed to predicting the high-risk population of stroke (**Figure 4B**). In addition, we utilized the same method to build a microbial diagnostic model I for IS and compared it with another diagnostic model II established with epidemiological data related to IS (**Supplementary Figure 2**). The AUC of model II increased to 81.8 (95% CI, 73.54%–90.01%; p < 0.01) compared with the AUC of 65.9% (95% CI, 55.11%–76.75%; p < 0.05) for model I (**Figure 4C**). Although an oral microbiome diagnosis is not required for IS, the high AUC for model II shows that the oral flora of patients with IS can differentiate HC with more sensitivity and specificity than epidemiological data.

### Relationship between oral microbiota and IS types

We divided patients with IS into LAA and SAA groups on the basis of TOAST criteria to explore whether the type of IS affects oral bacteria. The baseline data of subjects in each group are shown in **Supplementary Table 1**. The results indicated no differences in alpha and beta diversity analyses between the two groups (**Supplementary Figures 3A, B**). Furthermore, at the genus level, LEfSe analysis revealed a higher relative abundance of *g_Macellibacteroides*, *g_Pseudimonas*, *g_Ruminococcus*, *g_Caulobacter*, *g_Ralstonia*, and *g_Aquabacterium* in the LAA, whereas *g_Veillonella* was more abundant in the SAA (**Supplementary Figure 3C**).

### Relationship between oral microbiome and IS severity

**Figure 5:**
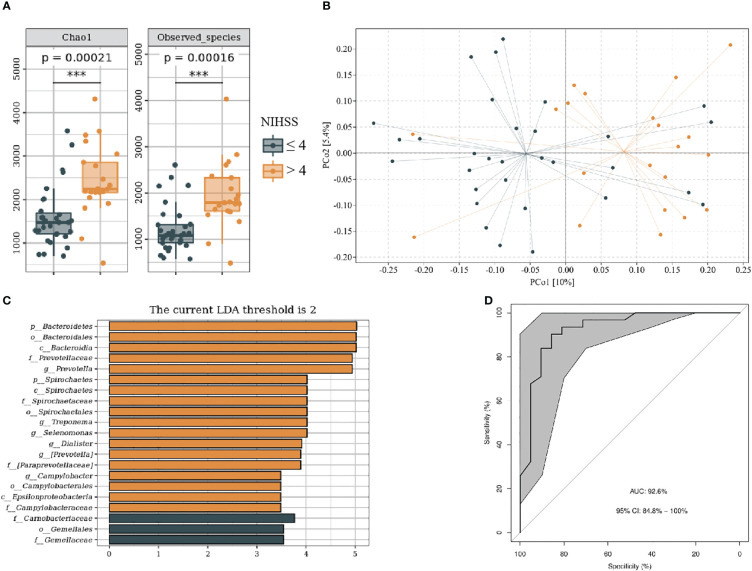
Oral microbiota composition of patients with NIHSS ≤ 4 and NIHSS > 4. **(A)** Comparison of α-diversity between the oral microbiota in two groups. **(B)** Principal coordinate analysis based on the unweighted UniFrac distances. **(C)** linear discriminant analysis effect size-determined differential features. **(D)** Machine learning for classification based on oral microbiota using the random forest algorithm. ***: P value ≤ 0.001.

We divided patients with IS into NIHSS score ≤ 4 and NIHSS score > 4 groups on the basis of neurological dysfunction on admission. The baseline data of subjects in each group are shown in **Supplementary Table 2**. The NIHSS > 4 group showed more alpha (P < 0.001) and beta (F = 2.19, P < 0.001) diversity (**Figures 5A, B**). LEfSe analysis indicated that the NIHSS > 4 had a greater relative abundance of *g_Prevotella*, *g_Treponema*, *g_Selenomonas*, *g_Dialister*, *g_Campylobacter*, and *g_Aquabacterium* (**Figure 5C**). We included genus levels with LDA scores > 2 in ROC analysis, and the model’s AUC is 92.6% (95% CI, 84.8%–100%; p < 0.01). In general, the model constructed by oral microbiota has a high diagnostic value for the severity of IS (**Figure 5D**).

### Relationship between oral microbiome and IS prognosis

**Figure 6:**
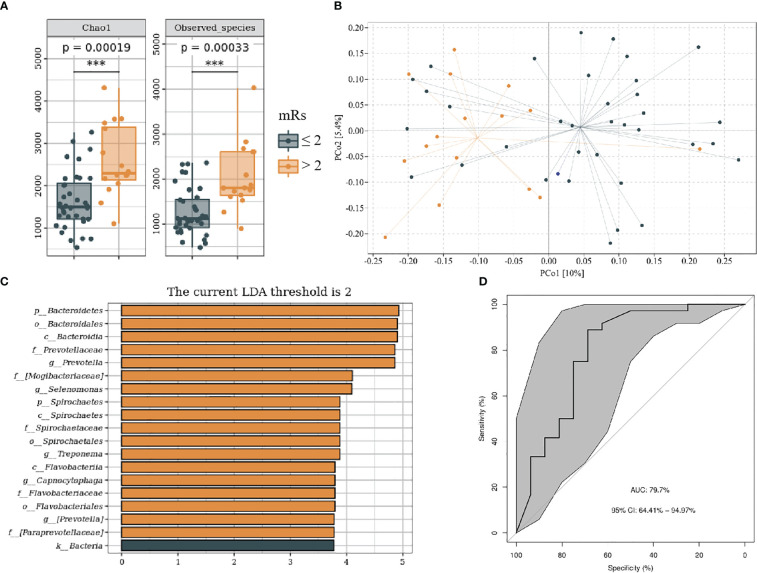
Oral microbiota composition of patients with mRs ≤ 2 and mRs > 2. **(A)** Comparison of α-diversity between the oral microbiota in two groups. **(B)** Principal coordinate analysis based on the unweighted UniFrac distances. **(C)** linear discriminant analysis effect size-determined differential features. **(D)** Machine learning for classification based on oral microbiota using the random forest algorithm. ***: P value ≤ 0.001.

We divided patients with IS into mRs score ≤ 2 and mRs score > 2 groups on the basis of functional outcome 90 days following stroke onset. The baseline data of subjects in each group are shown in **Supplementary Table 3**. Compared with mRs ≤ 2, the mRs > 2 groups showed higher alpha and beta (F = 1.31, P < 0.05) diversity (**Figures 6A, B**). LEfSe analysis revealed that *g_Prevotella*, *g_Selenomonas*, *g_Treponema*, and *g_Campylobacter* were more abundant in the mRs > 2 (**Figure 6C**). Furthermore, we constructed the predictive model by differential genera to effectively distinguish patients with IS with poor 90-day prognoses from those with good (**Figure 6D**; AUC = 79.7%; 95% CI, 64.41%–94.97%; p < 0.01).

### Relationship between oral microbiome and lipid metabolism

Spearman’s rank–based analysis investigated the relationship between blood lipid parameters and oral microbiota detected in all saliva samples. Our result shows that several oral bacteria show a high correlation with multiple lipid parameters, which may contribute to the pathogenesis of IS. In the genus level, *g_Prevotella* positively correlated with TC (r = 0.23, p < 0.01), TG (r = 0.37, p < 0.001), and LDL (r = 0.19, p < 0.05); *g_Fusobacterium* positively correlated with TC (r = 0.17, p < 0.05) and TG (r = 0.20, p < 0.05); *g_Leptotrichia* positively correlated with TC (r = 0.17, p < 0.05) and TG (r = 0.18, p < 0.05); and *g_Treponema* positively correlated with TC (r = 0.11, p < 0.05) and TG (r = 0.30, p < 0.001) and negatively correlated with HDL (r = −0.19, p < 0.05) (**Supplementary Figure 4**).

## Discussion

In this study, we depicted the oral salivary microbial profile of patients with IS and HRIS and constructed diagnostic models by differential genera to predict the severity and prognosis of IS. We observed that the diversity of the HRIS and IS saliva flora was considerably higher than that of HC, and the microbiota composition differed between the three groups.

Currently, the research on the relationship between stroke and human microbiomes mainly focuses on intestinal bacteria, with little attention paid to the oral microbiome. An observational study found that patients with stroke had gut microbiota dysregulation compared with healthy individuals (29). In animal models, transplantation of fecal bacteria from patients with stroke into germ-free mice aggravates ischemia-induced brain injury volume and functional deficits (30). However, several studies have shown that oral microbiomes have high homology with intestinal flora, and some oral bacteria can colonize the gastrointestinal tract (31–34). Ding and Schloss found that changes in oral bacteria can cause dysbiosis in the intestinal flora through the Dirichlet multinomial mixing model (35). Therefore, we explore the relationship between stroke and oral bacteria to help better understand human microorganisms’ role in cerebrovascular diseases. We found a high abundance of *g_Streptococcus* in all three groups, similar to the previous study by Boaden et al. (23) Compared with HC, the relative abundance of *g_Streptococcus*, *g_Prevotella*, *g_Veillonella*, and *g_Fusobacterium* was higher in HRIS and IS, and partial genera show an increasing trend. It can be assumed that these changes in the oral microbiome may have occurred in stroke-prone individuals before the actual onset of stroke. The intricate mechanisms at play are not yet clear, and the following are some probable interpretations. First, dysbiosis of oral flora may accelerate atherosclerosis. A meta-analysis found *Streptococcus mutans* in atherosclerotic plaque samples from patients, suggesting that oral bacteria may be transported into the circulation and then into the atherosclerotic plaque (36). Bacteria entering the blood can be recognized by microbe-associated molecular pattern–pattern recognition receptor signaling, which can accelerate atherosclerosis by stimulating the release of inflammatory cytokines (37). A clinical study reported that the high relative abundance of *g_Streptococcus* in the intestine is linked to atherosclerosis, which makes reason to hypothesize that oral bacteria can lead to gut flora dysregulation through ectopy (38). In addition, an increased abundance of some oral bacteria may cause metabolic disorders, which increase the risk of cerebrovascular diseases. Our results found that the abundance of *g_Fusobacterium* and *g_Prevotella* presented an increasing trend in the three groups. Recent studies have linked an increased abundance of *g_Fusobacterium* and *g_Prevotella* with atherosclerosis and metabolic disorders (39, 40). Zhou et al. found that *Fusobacterium* promotes ox-LDL–induced cholesterol phagocytosis and accumulation by regulating the expression of genes involved in lipid metabolism. In addition, it can invade aortic tissue and significantly increase the progression of atherosclerotic lesions (41). An animal experiment found that germ-free mice transplanted with Prevotella flora from patients with hypertension had higher blood pressure (42). In nondiabetic subjects, high *g_Prevotella* abundance was associated with obesity, BMI, insulin resistance, hypertension, and nonalcoholic fatty liver disease (43). Meanwhile, *Fusobacterium nucleatum*, *Prevotella intermedia*, and *Prevotella nigrescens* are closely associated with periodontitis infections. A cohort study followed up for 15 years demonstrated that periodontitis is an independent risk factor for ischemic stroke, and regular oral care reduces the incidence of stroke (13). Jaramillo et al. conducted a multicenter study that found that patients with periodontitis had higher TG and HDL levels, but the mechanisms involved must be further explored experimentally (44). We observed that *g_Fusobacterium* and *g_Prevotella* showed a strong positive correlation with TC and TG using Spearman analysis, which may play a role in linking periodontitis to lipid metabolism.

We also evaluated the severity and prognosis of IS by different saliva microbiomes. The result shows that the abundance of oral conditional pathogenic bacteria was significantly higher in patients with severe IS compared with those with mild IS and constructed diagnostic models by differentiated genera with high specificity and sensitivity. The underlying mechanism may be related to post-stroke infection. Stroke-related pneumonia was the leading cause of poor outcomes after stroke. Stroke-induced stress can cause immunosuppression and thus increase susceptibility to infection (45). An animal experiment verified that mice receiving intragastric administration of periodontitis salivary microbiota increased immune cell production of Interleukin-17A in the gut and eventually showed significantly worse stroke outcomes (46). Fabian et al. conducted a prospective study that found Neisseria, Porphyromonas, and Prevotella could be associated with patients with pneumonia, but no significant difference (47). Thus, more reliable evidence remains to be explored further.

The innovation of this study is that we revealed the oral microbiome status in patients with IS, HRIS, and HC by 16S rRNA sequence. Compared with HC, the oral bacteria of HRIS were also significantly different. Partially, genera had an increasing trend among the three groups, which predicted that oral bacteria could be a high-risk factor for stroke. In a follow-up of the neurological function of the IS, prognosis models constructed using oral microbiota have high predictive value. These results may be more helpful in understanding the role oral flora play in the development and progression of stroke. However, some of the following limitations also exist. First, this study included LAA and SAA types from the TOAST IS categorization. There was no noticeable difference in microbial diversity between the two groups. However, it cannot be ruled out that other types of stroke (cardiogenic cerebral embolism and ischemic stroke due to other causes) will affect diversity. Second, we only collected saliva samples from patients at admission and did not follow up dynamically on their oral microbiology at discharge or 1 month after onset. Therefore, we are unsure whether the microbiome dysbiosis in the IS oral will improve with stroke treatment. Third, the prognostic assessment of patients with IS in this study was insufficient, and the cognitive performance of the patients was not followed up. Several studies have confirmed the association between oral microbes and vascular cognitive impairment, and we will follow up on cognitive function in this group of patients in the future. Last, this study was a single-center, case-control study with a relatively small sample of subjects. The findings need to be validated in a multicenter, large-sample cohort study.

In summary, our finding reveals a significant alteration of oral flora in IS and IS high-risk patients, and the differential bacteria have some predictive value for the severity and prognosis of IS. Oral microbiota may be used as potential biomarkers in patients with IS.

## Data availability statement

The datasets presented in this study can be found in online repositories. The names of the repository/repositories and accession number(s) can be found below: PRJNA938448 (SRA).

## Ethics statement

The studies involving human participants were reviewed and approved by Ethics Committee for Human Experimentation of the Fifth People’s Hospital of Shanghai, China. Written informed consent to participate in this study was provided by the participants’ legal guardian/next of kin.

## Author contributions

Design: DW; Drafting: WS; Revising: DW and XY; Acquisition data: WS, SH, and LL; Analysis data: WS and YL. All authors contributed to the article and approved the submitted version.
