# Supplementary material for: The oral microbiome of patients with ischemic stroke predicts their severity and prognosis
Source: Front Immunol. 2023 Apr 17;14:1171898. doi: 10.3389/fimmu.2023.1171898 (PMC10150016; doi:10.3389/fimmu.2023.1171898)
Supplement: Supplementary file 1 [file DataSheet_1.pdf]

Supplementary table 1. Characteristics of the Study Participants

	SAA<br>n=28	LAA<br>n=24	P value
Age, years	56±7.5	58±8.3	0.192
Male, n (%)	19(67.9)	11(45.8)	0.160
BMI, kg/m2	26.6±3.6	27.0±3.3	0.705
Current smoking, n (%)	16(57.1)	6(25.0)	0.026
Hypertension, n (%）	21(75.0)	20(83.3)	0.463
Diabetes Mellitus, n (%）	11(39.3)	13(54.2)	0.403
Family history of stroke, n (%）	7(25.0)	2(8.3)	0.152
Triglycerides, mmol/L	1.95±0.92	1.60±0.74	0.196
Total cholesterol, mmol/L	4.81±1.03	4.84±1.42	0.139
HDL cholesterol, mmol/L	1.04±0.23	1.08±0.27	0.133
LDL cholesterol, mmol/L	2.84±1.03	2.74±1.29	0.939

Note: BMI = body mass index; LDL cholesterol = low density lipoprotein cholesterol; HDL cholesterol = high density lipoprotein cholesterol

Supplementary table 2. Characteristics of the Study Participants

	NIHSS≤ 4<br>n=31	NIHSS＞4<br>n=21	P value
Age, years	54±6.9	61±7.6	＜0.01
Male, n (%)	16(51.6)	14(66.7)	0.392
BMI, kg/m2	26.1±3.0	27.3±3.7	0.243
Current smoking, n (%)	13(41.9)	9(42.9)	0.947
Hypertension, n (%）	23(74.2)	18(85.7)	0.491
Diabetes Mellitus, n (%）	16(51.6)	8(38.1)	0.403
Family history of stroke, n (%）	5(16.1)	4(19.0)	0.787
Triglycerides, mmol/L	1.80±0.91	1.77±0.79	0.907
Total cholesterol, mmol/L	4.66±1.16	5.08±1.29	0.235
HDL cholesterol, mmol/L	1.07±0.26	1.03±0.23	0.658
LDL cholesterol, mmol/L	2.72±1.17	2.91±1.14	0.575

Note: BMI = body mass index; LDL cholesterol = low density lipoprotein cholesterol; HDL cholesterol = high density lipoprotein cholesterol

Supplementary table 3. Characteristics of the Study Participants

	mRS≤ 2<br>n=36	mRS > 2<br>n=16	P Value
Age, years	55±7.0	61±8.3	0.012
Male, n (%)	15(58.3)	9(56.3)	0.888
BMI, kg/m2	26.6±2.7	26.9±3.7	0.774
Current smoking, n (%)	17(47.2)	5(31.3)	0.368
Hypertension, n (%）	27(75.0)	14(87.5)	0.468
Diabetes Mellitus, n (%）	16(44.4)	8(50.0)	0.769
Family history of stroke, n (%）	6(16.7)	3(18.8)	0.855
Triglycerides, mmol/L	1.83±0.91	1.70±0.71	0.575
Total cholesterol, mmol/L	4.66±1.14	5.19±1.34	0.235
HDL cholesterol, mmol/L	1.03±0.26	1.10±0.20	0.357
LDL cholesterol, mmol/L	2.66±1.12	3.10±1.19	0.213

Note: BMI = body mass index; LDL cholesterol = low density lipoprotein cholesterol; HDL cholesterol = high density lipoprotein cholesterol

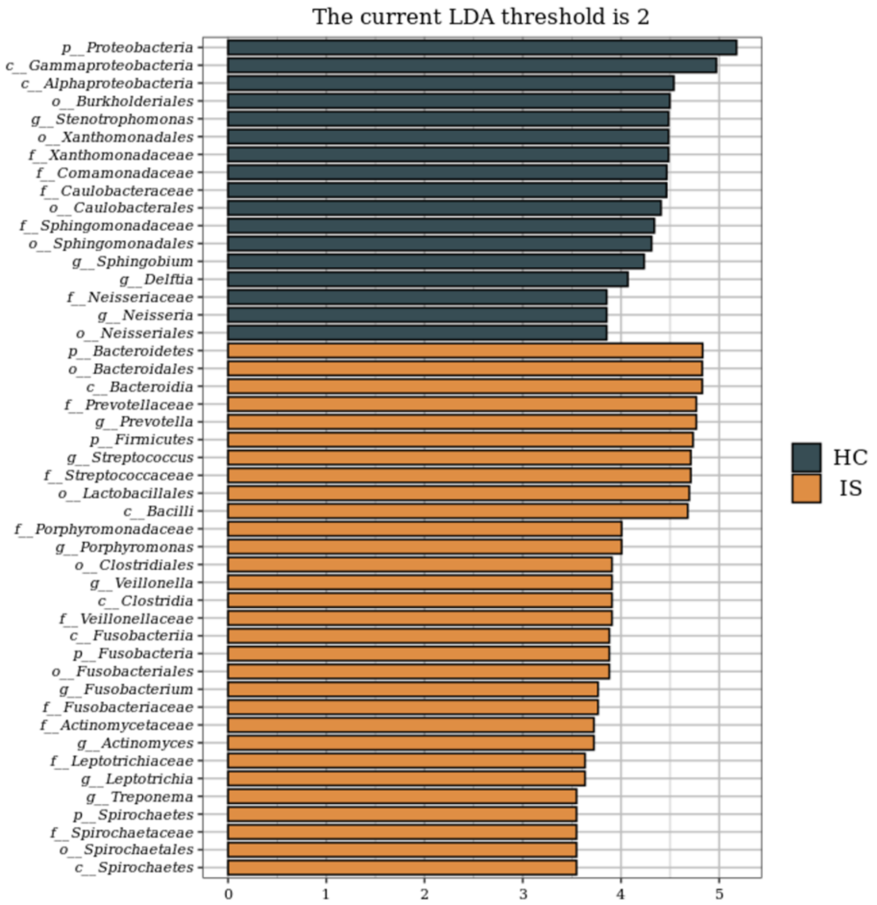

Supplementary figure 1: Difference of oral microorganisms between HC and IS

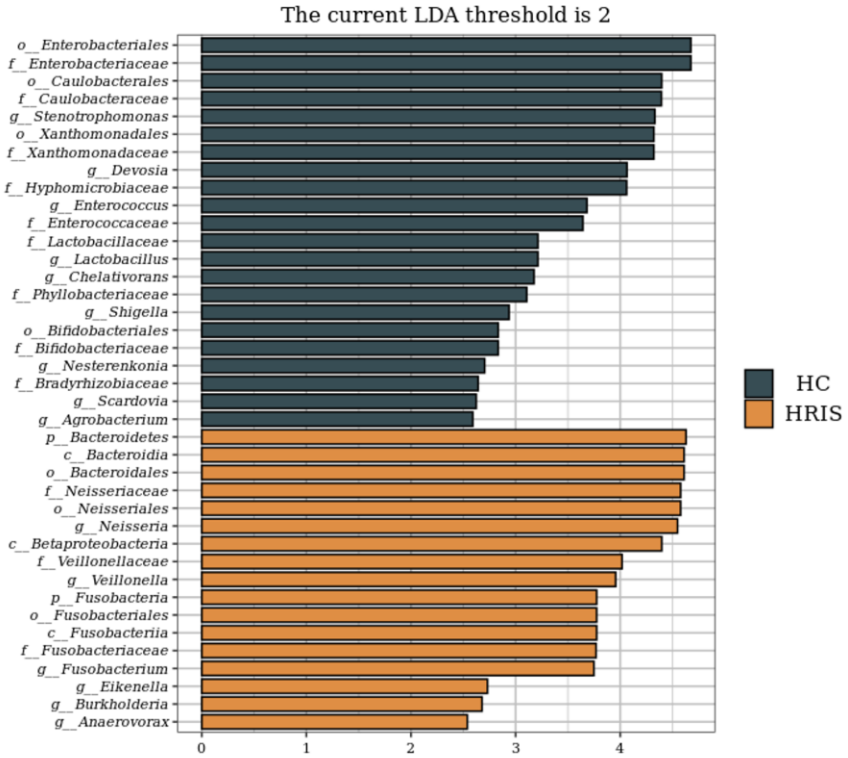

Supplementary figure 2: Difference of oral microorganisms between HC and HRIS

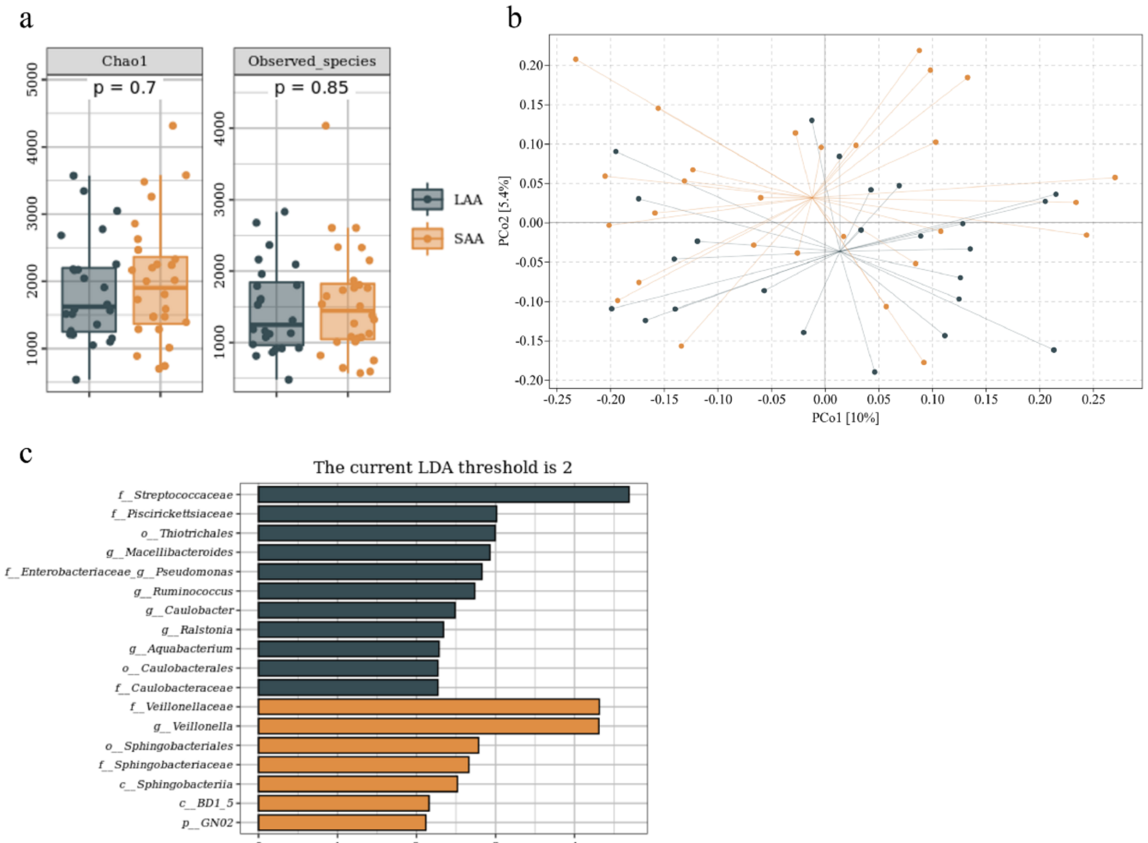

Supplementary figure 3:Oral microbiota composition of patients with SAA and LAA. a) Comparison of α-diversity between the oral microbiota in two groups. b) Principal coordinate analysis based on the unweighted UniFrac distances. c) linear discriminant analysis effect size–determined differential features.

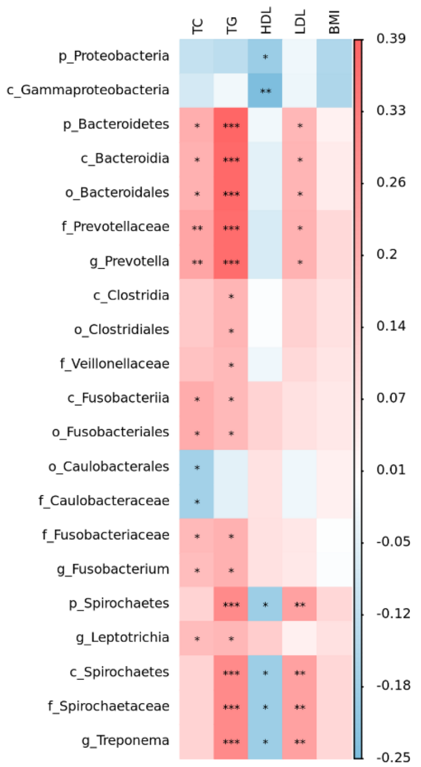

Supplementary figure 4: Association of microbiota and the lipid metabolism
